# Antibiograms, multidrug resistance, and milk-related parameters of bacteria isolated from milk of dairy cattle in Phatthalung, Thailand

**DOI:** 10.14202/vetworld.2024.735-743

**Published:** 2024-04-03

**Authors:** Supaporn Somrup, Watcharapong Mitsuwan, Teeraphun Bhumibhamon, Maria de Lourdes Pereira, Alok K. Paul, Veeranoot Nissapatorn, Phirabhat Saengsawang

**Affiliations:** 1 Faculty of Technology and Community Development, Thaksin University, Phatthalung 93210, Thailand; 2 Akkhraratchakumari Veterinary College, Walailak University, Nakhon Si Thammarat 80160, Thailand; 3 One Health Research Center, Walailak University, Nakhon Si Thammarat 80160, Thailand; 4 Center of Excellence in Innovation of Essential Oils, Walailak University, Nakhon Si Thammarat 80160, Thailand; 5 Veterinary Research and Development Center (Upper Southern Region), Nakhon Si Thammarat 80110, Thailand; 6 Department of Medical Sciences, CICECO-Aveiro Institute of Materials, University of Aveiro, Aveiro 3810-193, Portugal; 7 School of Pharmacy and Pharmacology, University of Tasmania, Hobart, Australia; 8 School of Allied Health Sciences, Southeast Asia Water Team, World Union for Herbal Drug Discovery, and Research Excellence Center for Innovation and Health Products, Walailak University, Nakhon Si Thammarat 80160, Thailand

**Keywords:** antibiotic susceptibility, dairy cattle, milk composition, milk quality, Thailand

## Abstract

**Background and Aim:**

Milk, a nutritious food, is widely consumed in human diets; however, contamination by micro-organisms can negatively impact its quality and consumer health. Contamination by micro-organisms affects the quality of milk, which can affect the quality of the milk production chain. This study aimed to determine the changes in milk composition and antibiotic susceptibility related to bacteria isolated from dairy cow milk.

**Materials and Methods:**

Raw milk samples were collected from 72 dairy cows. All milk samples were subjected to the California Mastitis Test (CMT) for CMT score determination. We also investigated milk composition, bacterial culture (BC), and antibiotic susceptibility.

**Results:**

About 47.22% and 30.56% of dairy cattle were positive for CMT + BC and automatic somatic cell count (ASCC) + BC, respectively. Fecal appearance and animal age were found to be risk factors for ASCC + BC positivity in dairy cattle. Bacteria were found in approximately 76% of milk samples, with the most common isolated species being α-hemolytic *Streptococcus* spp., coagulase-negative *Staphylococcus* spp., and *Escherichia coli*. Of these, 70% are resistant to at least one antibiotic. Variation in the multidrug resistance pattern was high in *Klebsiella* spp.

**Conclusions:**

Fecal appearance and animal age are risk factors for ASCC + BC positivity in dairy cattle. This study identified antibiotic and multidrug resistance patterns, which require comprehensive studies and effective surveillance systems. Remarkably, the use of antibiotic therapy in dairy cattle should be monitored.

## Introduction

Milk is a nutrition-rich food used in several menus of human diets, especially in low- and middle-income countries [1]; however, contamination by micro-organisms affects its quality. In addition, micro-organism growth in milk may affect consumer health [2]. Several factors, such as farm management and the hygienic aspect of milk collection, affect milk quality at the farm level. Good agricultural practices, particularly the prevention of microbial contamination of raw milk at the farm level, are critical aspects of the milk production industry. In addition, micro-organism contamination of raw milk affects the quality of the dairy milk production chain [3]. Contamination of micro-organisms in collected milk at the farm level originates from the external surface of the udder and teat, milking equipment, and mastitis-causing organisms inside the udder [4]. Milk quality is evaluated using several analyses, including milk composition analysis and microbiological methods.

Milk quality is related to udder health, and milk somatic cell count (SCC) is used to monitor the quality of dairy milk at the farm. In the milk industry, the hygienic aspects of milk production [5] control the quality and safety of raw milk. Bacterial contamination in milk is associated with milk quality worldwide [6] because micro-organisms cause changes in certain aspects of milk quality [7]. Lactobacilli, Streptococci, Enterococci, *Pseudomonas*, *Acinetobacter*, Staphylococci, *Listeria*, and *Salmonella* are common bacteria found in milk [8–10]. Interestingly, some bacteria can induce several food-borne diseases in humans and potentially risk human health [11].

Antibiotics are primarily used to treat diseases in dairy cattle, especially bovine mastitis; however, some farms use antibiotics as a preventative measure [12]. Moreover, reduction in antibiotic use has been promoted globally [13], and antimicrobial resistance, particularly antibiotic resistance (ABR), is a global concern [14]. Remarkably, ABR in mastitis-causing bacteria is a source of concern [15]. The main cause of ABR in the dairy industry is the incorrect use of antibiotic doses, and ABR in mastitis medication has been identified as an important aspect of One Health Practice [16]. In addition, ABR has been promoted as a public health concern, particularly for food chain production [17], and monitoring of ABR in the dairy industry is necessary [18].

The Department of Livestock Development of the Ministry of Agriculture and Cooperatives of Thailand revealed that the country would have approximately 810,000 dairy cattle in 2021–2022 [19]. Phatthalung province has the highest proportion of dairy cattle (77.64%) in the southern region of Thailand [19]. Thailand has a high temperature and relative humidity [20]. In addition, high ambient temperatures increase the risk of high SCCs [21] and easily induce the growth of micro-organisms in milk [22], which may adversely affect milk quality. However, the assessment of milk-contaminated bacteria related to milk composition and antibiograms in southern Thailand is limited.

This study aimed to determine the antibiograms and milk composition of milk-contaminated bacteria in dairy cattle in Phatthalung Province, southern Thailand.

## Materials and Methods

### Ethical approval

The Institutional Animal Care and Use Committee of Thaksin University approved the dairy cattle restraint and milk collection protocol for this study (approval ID: COA No. TSU2021-009/IACUC No. 0001). Milk collection and animal restraint were performed under the supervision of a veterinarian. All microbiological assays were performed at a certified private laboratory.

### Study period and location

This study was a cross-sectional study conducted from January 2022 to June 2022 at dairy cattle farms in Phatthalung province, Thailand.

### Studied population

The milk samples collected from dairy cattle in this study were divided into the following categories: (1) dairy milk samples (DC_CMT_) that were positive only for the California Mastitis Test (CMT); (2) dairy milk samples (DC_CMT+BC_) that were positive for both the CMT and bacterial culture (BC); (3) dairy milk samples (DC_ASCC_) that were positive only for the automated SCC (ASCC; result >200,000 cells/mL) without any bacterial growth; and (4) dairy milk samples (DC_ASCC+BC_) that were positive for both the ASCC (result >200,000 cells/mL) and BC.

### Target population and sample size

The sample size calculation formula used in this study is presented below. Calculations were based on previous prevalence (p) [23] and the number of dairy cattle population (N) [19, 24] in the study area. A total of 72 dairy cattle were calculated using the ProMESA version 2.3.0.2 program (EpiCentre, Massey University, New Zealand) developed by the EpiCentre of Massey University [25].


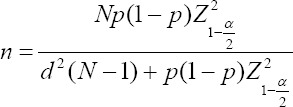


Where is:

p = Previous prevalence of bacterial contamination in milk sample.

Np = Number of dairy cattle population in the study area.

d = Maximum tolerated error from a similar study.

α = Alpha error.

### Characteristics of the studied dairy cattle

The characteristics of each dairy cow were recorded during milk collection. Veterinarians examined the external appearance of each dairy cattle. Examinations for body condition score (BCS), feces score (FS), and teat end score (TES) were performed. BCS (1–5), FS (1–4), and TES (no ring [N], smooth or slightly rough ring [S], rough ring [R], and very rough ring [VR]) scoring systems were used in accordance with previous publications [26–28].

### Milk sample collection

Teat disinfection was used to clean the udders of each dairy cow before milking. All teats were washed using a commercial udder washing solution containing chlorhexidine gluconate. Milk samples were collected manually from all quarters of the dairy cattle and placed in sterile centrifuge plastic tubes (10–20 mL of each pooled milk sample per dairy cattle). Milk samples were immediately placed on ice and submitted to laboratory units for BC, antibiotic sensitivity testing, and milk composition analysis.

### Screening of milk quality using CMT

CMT was used immediately after milk collection to screen individual milk samples. Briefly, 3% sodium lauryl sulfate and bromocresol were added to the collected milk sample, and the protocol for CMT used in this study was consistent with that used in the previous studies by Bhutto *et al*. [29]. According to the appearance of milk gel formation [30], the CMT results were classified as negative (N), trace (T), weakly positive (+1), or distinctly positive (+2).

### BC and antibiotic susceptibility testing using disk diffusion

Each milk sample was microbiologically analyzed to determine the type of bacteria using agar plate cultures and biochemical tests. Briefly, each milk sample was spread on blood agar (HiMedia Laboratories, Mumbai, India) and MacConkey agar (HiMedia Laboratories) and incubated at 37°C for 18–24 h. All pure culture isolates were stained with Gram stain. In addition, hemolysis patterns (α, β, and γ) of Gram-positive bacteria were observed on blood agar, and isolated bacteria were classified by biochemical tests. Gram-positive bacteria were re-cultured on mannitol salt agar (HiMedia Laboratories), and biochemical oxidase, catalase, and coagulase tests were performed. Edwards medium and brain heart infusion with 6.5% NaCl (HiMedia Laboratories) were used to identify suspected *Enterococcus* colonies. For Gram-negative bacteria, ten biochemical tests, including oxidase, catalase, triple sugar iron, Simmon’s citrate, indole, methyl red, Voges–Proskauer, urease, oxidative–fermentative, and motility tests, were performed. Pure bacterial colonies were then introduced using disk diffusion to determine antibiotic susceptibility. Single pure colonies were placed in Mueller–Hinton broth (HiMedia Laboratories) adjusted to a turbidity of 0.5 McFarland. On Mueller–Hinton agar (HiMedia Laboratories), ten antibiotic disks (HiMedia Laboratories; and Oxoid™, Thermo Fisher Scientific, Ely, United Kingdom), including amoxicillin 10 μg (AML), cephalexin 30 μg (CEP), chloramphenicol 30 μg (CHL), enrofloxacin 5 μg (ENR), erythromycin 15 μg (ERY), gentamicin 10 μg (GEN), oxytetracycline 30 μg (OTC), penicillin G 10 μg (PEN), sulfamethoxazole-trimethoprim 25 μg (SXT), and vancomycin 30 μg (VAN), were used to test antibiotic susceptibility; however, only the antibiotic drugs recommended by the Clinical and Laboratory Standards Institute (CLSI), the European Committee on Antimicrobial Susceptibility Testing (EUCAST), and the British Society for Antimicrobial Chemotherapy (BSAC) standardized disk susceptibility testing method (BSAC) were tested for each isolate. The diameter of the inhibition zone was measured and interpreted according to CLSI [31], EUCAST [32], and BSAC [33]. ENR for *Escherichia coli* and *Pseudomonas aeruginosa* and OTC for *Acinetobacter* spp., *E. coli*, *Klebsiella* spp., *Enterobacter* spp., and *Enterococcus* spp. were based on the previous studies by Benedict *et al*. [34], Pintarić *et al*. [35], Oliveira *et al*. [36], and Huang *et al*. [37].

### Milk composition analysis

All raw milk samples were analyzed for fat (% fat), protein (% protein), lactose (% lactose), non-fat milk solids (% SNF), total solids (%), and (SCC × 1000 cells). Fourier-transform infrared spectroscopy was used to examine a collection of milk compositions, except SCC (MilkoScan FT6000®, Foss Electric A/S, Hillerod, Denmark). The SCC of milk samples was also analyzed using an automated somatic cell counter (Fossomatic™ 5000 basic, Foss Electric). All analyzed milk compositions were compared with a reference raw milk quality standard (Thai Agricultural Standard; TAS 6003-2010) and are presented in Table-1.

**Table-1:** Raw milk quality standard (Thai Agricultural Standard; TAS 6003-2010).

Milk composition	Range of passed value	Unit
Fat	>3.35	%w/w
Protein	>3.00	%w/w
Lactose	Not included	%w/w
Milk solids not fat	>8.25	%w/w
Total solid	Not included	%w/w
Somatic cell count	<500	×1000 cells/mL

### Statistical analysis

We recorded all data using Microsoft® Excel 2019 (Microsoft Corporation, Redmond, Washington, USA). Descriptive statistics were used to analyze the collected data. The Mann–Whitney U test, Kruskal–Wallis test, Chi-square test, and Fisher’s exact test were used to analyze individual dairy cattle factors and microbiological results, depending on the type of data. In addition, only significant factors from the univariate analysis were subjected to multiple comparisons using Bonferroni correction. All statistical analyses were performed using the R programming language version 4.1.2 [38] with a 95% confidence interval, and p < 0.05 was considered significant.

## Results

### Characteristics of the studied dairy cattle

Approximately 40% of the dairy cattle studied had 3/5 BCS and 3/4 FS, and almost 70% of them were adult dairy cattle. Most dairy cattle had a rough to extremely rough teat end appearance for the TES, and the details of the dairy cattle are presented in Table-2. The percentages of fat, lactose, and SNF were significantly different (p < 0.05) among the milk composition parameters.

**Table-2:** Number of studied dairy cattle classified by animal factors.

Factors	Total n (%)
Age group of dairy cattle	
1–5 years	50 (69.44)
>5 years	22 (30.56)
Body condition score	
<3	21 (29.17)
3	46 (63.89)
>3	5 (6.94)
Feces score	
<3	21 (29.17)
3	32 (44.44)
>3	19 (26.39)
Teat end score	
N	11 (15.28)
S	17 (23.61)
R	33 (45.83)
VR	11 (15.28)
California Mastitis test score	
N	23 (31.94)
T	31 (43.06)
+1	15 (20.83)
+2	3 (4.17)

Teat end score includes N=No ring, S=Smooth or slightly rough ring, R=Rough ring, VR=Very rough ring, California Mastitis test score includes N=Negative, T=Trace, +1=Weakly positive, +2=Distinctly positive

### Proportion of bacterial contamination in milk and factors associated with milk quality

Approximately 68% of the milk samples showed gel formation, as detected by CMT. In addition, the proportion of serial detection of CMT and BC (DC_CMT+BC_) was 47.22%, and there was no correlation between the occurrence of DC_CMT_ and DC_CMT+BC_ (Z = 1.65; p = 0.95, κ = 0.24; 0.52–0.05). Interestingly, the proportion of DC_ASCC+BC_ in this study was 30.56%, and approximately 26% of DC_CMT+BC_ was DC_ASCC+BC_. Table-3 presents the proportions of DC_CMT+BC_ and DC_ASCC+BC_ classified by associated factors. The fecal appearance proportion significantly differed between the DC_ASCC+BC_ and non-DC_ASCC+BC_ groups (p < 0.05). In this study, a watery fecal appearance was associated with an approximately 6-fold increased risk of DC_ASCC+BC_ compared with a hard fecal appearance. Moreover, in this study, age was found to be a risk factor related to DC_ASCC+BC_. However, other animal factors did not differ between the DC_ASCC+BC_ and non-DC_ASCC+BC_ groups. However, approximately 20% and 26% of the DC_ASCC+BC_ group passed the standard quality cutoffs for protein and SNF in raw milk composition, respectively. Only approximately 5% of DC_ASCC+BC_ patients passed the standard cutoff for fat quality. Table-4 compares milk composition and DC_ASCC+BC_ in individual dairy cattle.

**Table-3:** Prevalence and 95% confidence interval of DC_CMT+BC_ and DC_ASCC+BC_ classified by animal factors.

Factor	DC_CMT+BC_		DC_ASCC+BC_	
	
	Prevalence (P/T, %)	95% CI	Prevalence (P/T, %)	95% CI
Cattle age				
1–5 years	24/50 (48.00)	33.66–62.58	7/50 (14.00)	5.82–26.74
>5 years	10/22 (45.45)	24.39–67.79	15/22 (68.18)	45.13–86.14
Body condition score				
<3	8/21 (38.10)	18.11–61.56	8/21 (38.10)	18.11–61.56
3	24/46 (52.17)	36.95–67.11	14/46 (30.43)	17.74–45.75
>3	2/5 (40)	5.27–85.34	0/5 (0.00)	–
Feces score				
<3	12/21 (57.14)	34.02–78.18	3/21 (14.29)	3.05–36.34
3	18/32 (56.25)	37.66–73.64	9/32 (28.13)	13.75–46.75
>3	4/19 (21.05)	6.05–45.57	10/19 (52.63)	28.86–75.55
Teat end score				
N	6/11 (54.55)	23.38–83.25	1/11 (9.09)	0.23–41.28
S	9/17 (52.94)	27.81–77.02	6/17 (35.29)	14.21–61.67
R	17/33 (51.52)	33.54–69.20	14/33 (42.42)	25.48–60.78
VR	2/11 (18.18)	2.28–51.78	1/11 (9.09)	0.23–41.28
Total	34/72 (47.22)	35.33–59.35	22/72 (30.56)	20.24–42.53

P=Positive, T=Total, CMT=California mastitis test, BC=Bacterial culture, ASCC=Automated somatic cell count, N=No ring, S=Smooth or slightly rough ring, R=Rough ring, VR=Very rough ring, CI=Confidence interval

**Table-4:** Animal and milk composition factors related to DC_ASCC+BC_ at the individual dairy cattle level.

Factor	DC_ASCC+BC_		p-value	OR (95% CI)

	Yes	No		
Individual factor				
Gram				
Positive	15	15	0.18^a^	-
Negative	10	3		
Age group				
1–5 years	7	43	<0.05^b^	13.16 (3.96–43.76)
>5 years^ref^	15	7		
BCS				
<3	8	13	0.30^a^	-
3	14	32		
>3	0	5		
FS				
<3	3	18	<0.05^a^	FS < 3 compared with reference 0.15 (0.03–0.68)
3	9	23		
>3^ref^	10	9		
TES				
N	1	10	0.07^a^	-
S	6	11		
R	14	19		
VR	1	10		
Milk composition				
Fat				
Passed	4	3	0.21^a^	-
Failed	18	42		
Protein				
Passed	18	31	0.38^a^	-
Failed	4	14		
SNF				
Passed	14	35	0.22^b^	-
Failed	8	10		

SNF=Solids-not-fat, BCS=Body condition score, FS=Feces score, TES=Teat end score, N=No ring, S=Smooth or slightly rough ring, R=Rough ring, VR=Very rough ring, a=tested by Fisher’s Exact test, b=tested by Chi-square test

### Isolation and antibiotic susceptibility of milk samples

Approximately 76% (55/72) of the milk samples collected contained at least one species of bacteria. Gram-positive and-negative bacteria were coagulase-negative *Staphylococcus* spp. (CNSt) and *E. coli*, respectively. Of the bacteria isolated from milk samples, 47.27% (26/55) and 70.91% (39/55) were coevident (Table-5) and resistant to antibiotics (Table-6), respectively. GEN- and SXT-resistant bacteria were the most prevalent in raw milk, and *Klebsiella* spp. (50%) and *Enterobacter* spp. (20%) were identified as the main antibiotic-resistant specie. There was no difference in the milk composition and Gram-type bacteria in the dairy cattle studied. Protein and SNF compositions were significantly different between susceptible and resistant bacteria (p < 0.05). Antibiotics that act on cell walls and nucleic acid syntheses were found to be the main multidrug-resistant (MDR) group in this study (Table-7); however, no specific MDR pattern was observed. In addition, an isolate of *Enterobacter* spp. was resistant to all tested antibiotics with an MDR pattern (AML-CEP-CHL-GEN-OTC-SXT) according to the full names of these antibiotics.

**Table-5:** Bacterial culture results of individual raw milk samples classified by the number of isolated bacterial species.

Bacterial contamination	n (%)
Single contamination (n = 29)	
*Klebsiella* spp.	1 (3.45)
*Acinetobacter* spp.	2 (6.90)
*Enterococcus* spp.	3 (10.34)
α hemolytic *Streptococcus* spp.	6 (20.69)
*Escherichia coli*	8 (27.59)
Coagulase negative *Staphylococcus* spp.	9 (31.03)
Co-contamination (n = 26)	
*Escherichia coli* + α hemolytic *Streptococcus* spp.	1 (3.85)
Coagulase negative *Staphylococcus* spp. + *Escherichia coli*	1 (3.85)
α hemolytic *Streptococcus* spp. + *Klebsiella* spp.	1 (3.85)
*Acinetobacter* spp. + *Klebsiella* spp.	1 (3.85)
*Enterococcus* spp. + *Klebsiella* spp.	1 (3.85)
*Enterobacter* spp. + *Enterococcus* spp.	1 (3.85)
*Enterobacter* spp. + β hemolytic *Streptococcus* spp.	1 (3.85)
Coagulase negative *Staphylococcus* spp. + *Pseudomonas* spp.	1 (3.85)
*Acinetobacter* spp. + *Bacillus* spp.	1 (3.85)
*Enterobacter* spp. + *Escherichia coli*	1 (3.85)
*Acinetobacter* spp. + *Escherichia coli*	1 (3.85)
*Bacillus* spp. + Coagulase negative *Staphylococcus* spp.	2 (7.69)
Coagulase negative *Staphylococcus* spp. + *Enterobacter* spp.	3 (11.54)
Coagulase negative *Staphylococcus* spp. + *Enterococcus* spp.	3 (11.54)
Coagulase negative *Staphylococcus* spp. + α hemolytic *Streptococcus* spp.	7 (26.92)

**Table-6:** Antibiotic susceptibility results of milk sample contaminated with bacteria.

Isolated bacteria	AML	CEP	CHL	ENR	ERY	GEN	OTC	PEN	SXT	VAN	MDR (%)
AC	-	-	-	-	-	2	0	-	2	-	0/5 (0)
αHSt	1	-	0	-	0	7	-	1	2	4	1/14 (7.14)
βHSt	-	-	0	-	0	1	-	0	0	0	0/2 (0)
CNSt	-	-	0	-	9	-	-	7	6	-	3/26 (11.54)
Ec	2	1	0	0	-	0	2	-	1	-	1/12 (8.33)
Klp	4	1	1	-	-	0	2	-	2	-	2/4 (50.00)
Etb	2	1	1	-	-	1	1	-	4	-	1/5 (20.00)
Etc	0	-	0	-	-	-	2	0	0	0	0/9 (0)
Ps	-	-	-	0	-	0	-	-	-	-	0/1 (0)
Ba	-	-	-	-	0	-	-	-	-	-	0/3 (0)
Total	9/44 (20.45)	3/21 (14.29)	2/72 (2.78)	0/13 (0)	9/45 (20.00)	11/43 (25.58)	7/35 (20.00)	8/51 (15.69)	17/77 (22.08)	4/25 (16.00)	

AC=*Acinetobacter* spp., aHst=a hemolytic *Streptococcus* spp., Ba=*Bacillus* spp., bHst=b hemolytic *Streptococcus* spp., CNSt=Coagulase negative *Staphylococcus* spp., Ec=*Escherichia coli*, Klp=*Klebsiella pneumoniae*, Klsp=*Klebsiella* spp., Etb=*Enterobacter* spp., Etc=*Enterococcus* spp., Ps=*Pseudomonas aeruginosa*, AML=Amoxicillin, CEP=Cephalexin, CHL=Chloramphenicol, ENR=Enrofloxacin, ERY=Erythromycin, GEN=Gentamicin, OTC=Oxytetracycline, PEN=Penicillin G, SXT=Sulfamethoxazole-trimethoprim, VAN=Vancomycin

**Table-7:** MDR patterns of Gram positive and Gram-negative bacteria isolated from raw milk samples.

Gram	Bacteria	MDR pattern	n	Cell Wall synthesis (n=51)			Nucleic acid synthesis (n=15)		Protein synthesis (n=44)			
		
				BL		VA	FL	DG	30s		50s	
					
				Penicillin	Cephalosporins	Vancomycin	Sulfonamides- Trimethoprim	Quinolones	Tetracyclines	Aminoglycosides	Macrolides	Chloramphenicol
Positive	αHSt	AML-GEN - PEN-SXT	1	R	-	S	R	-	-	R	S	S
	CNSt	ERY-PEN-SXT	1	R	-	-	R	-	-	-	R	S
Negative	EC	AML-OTC-SXT	1	R	S	-	R	R	S	S	-	S
	Etb	AML-CEP- CHL-GEN - OTC-SXT	1	R	R	-	R	-	R	R	-	R
	Klp	AML-CHL - OTC-SXT	1	R	S	-	R	-	R	S	-	R
		AML-CEP - OTC-SXT	1	R	R	-	R	-	R	S	-	S

BL=Beta-lactams, VA=Vancomycin, FL=Folate synthesis, DG=DNA gyrase, 30s=30s subunit, 50s=50s subunit, AML=Amoxicillin, CEP=Cephalexin, CHL=Chloramphenicol, ENR=Enrofloxacin, ERY=Erythromycin, GEN=Gentamicin, OTC=Oxytetracycline, PEN=Penicillin G, SXT=Sulfamethoxazole–trimethoprim, VAN=Vancomycin, R=Resistant, S=Susceptible, dash=Not tested

## Discussion

It is estimated that approximately 68% of dairy cattle show an abnormal milk reaction using the CMT technique. This study found that the proportion of DC_CMT_ in individual dairy cattle was similar to that found in Indonesia [39]; however, in contrast to other studies in Tunisia [40], Ethiopia [41, 42], and Tanzania [43], this rate was different. The proportion of DC_CMT_ in this study was high, and various risk factors, including housing, bedding, farm hygiene, mastitis history, milking method, milking machine cleanliness, and udder health monitoring, have been identified elsewhere [44–47]. In addition, approximately 4% of DC_ASCC_ milk samples were negative for bacteria. The culture of DC_ASCC_ milk samples without bacteria may have occurred due to infection with other microbes, such as yeast, mycoplasma, or fungi, which triggered the proliferation of immune cells invading udders and resulted in several somatic cells in the milk [43]. However, this event may have occurred due to a short-term infection of bacteria before sample collection or pathogen removal due to the immunity of dairy cattle [48].

In the present study, FS of dairy cattle was identified as a risk factor for DC_ASCC+BC_, and the watery appearance of feces was associated with a higher proportion of DC_ASCC+BC_ than other conditions. According to a study on the relationship between fecal consistency and bovine mastitis [49], loose feces spill directly onto the legs, tails, and udders, resulting in fecal contamination. In addition, sanitary practices are risk factors for subclinical mastitis associated with fecal consistency [49]. BCS and TES, on the other hand, were not identified as risk factors for DC_ASCC+BC_ in this study. A study by Birhanu *et al*. [47] also supported that BCS in cattle was not a risk factor for DCASCC+BC.

Our findings further showed that approximately 76% of the bacteria were isolated from raw milk samples, which is similar to other studies in China [50], Algeria [51], and Tanzania [43]. However, the type of isolated bacteria in milk differed significantly according to the characteristics of the study site. Most of the bacteria isolated in this study were environmental bacteria, which is in agreement with the previous studies by Song *et al*. [50] and Ruegg [52]. BC is a potential diagnostic approach for subclinical mastitis [53]; however, the disparity in positive bacteriology rates may reflect regional differences in disease treatment and control programs. A high rate of two isolated bacterial species was found in this study, similar to a study conducted in Egypt [44]. Interestingly, our study revealed that *Staphylococcus* spp., *Streptococcus* spp., *E. coli*, and *Enterococcus* spp. were the most prevalent bacteria isolated from raw milk, similar to a previous study conducted in Romania [54]. CNSt was found to be the most prevalent in raw milk in this study. CNSt is one of the most common mastitis-producing bacteria in dairy cattle, causing persistent infection [55]. In addition, CNSt affects milk quality [56]. *Streptococcus* spp., mainly α hemolytic species, was also found in high proportions in our recruited dairy cattle. *Streptococcus uberis* is the most common α-hemolytic *Streptococcus* species. [57]; however, some uncommon species, such as *Streptococcus lutetiensis*, have been isolated [57]. *E. coli* has been defined as an important environmental pathogen of subclinical mastitis [44], and the high rate of environmental pathogens may result from improper hygienic management of the farm. *E. coli* was the most prevalent Gram-negative bacterium found in raw milk samples. Notably, both CNSt and *E. coli* are concerned regarding ABR in the dairy industry and public health [54, 58].

Approximately 70% of the bacteria identified in milk samples are resistant to at least one class of antibiotics. At present, ABR in the dairy industry is an extremely concerning problem. Remarkably, the misuse of antibiotics for the treatment of mastitis has been mentioned as a major issue of ABR in dairy cattle [59, 60]. GEN and SXT were found to be the most prevalent ABR antibiotics in this study. In addition, several studies have identified bacteria resistant to GEN, including *Streptococcus* spp. [44], *Staphylococcus aureus* [61], *E. coli* [12], and *Klebsiella* spp. [62]. Our data showed high ABR in *Klebsiella* spp., *Enterobacter* spp., and CNSt. Prolonged and incorrect use of antibiotics is a probable cause of ABR in *E. coli* [63]. *Staphylococcus* spp. ABR is related to the formation of biofilms [64]. In this study, the penicillin and sulfonamide groups were identified as major drug members with various MDR patterns. In addition, amoxicillin and SXT were identified as the main drug members in several patterns of MDR. MDR was extremely concerned about the dairy industry [43]. Furthermore, MDR has been promoted as a global issue in veterinary medicine and public health [43]. Remarkably, food chain contamination with ABR bacteria has been recognized as a critical issue affecting the success of disease treatment [65]. In this study, *Klebsiella* spp. was found to have a high prevalence of MDR. *Klebsiella* spp. has also been identified as a significant MDR species elsewhere [65], [67].

## Conclusion

Fecal appearance and animal age were identified as potential risk factors for DC_ASCC+BC_, and approximately 20% of dairy cattle with DC_ASCC+BC_ passed the standard of milk composition, except for fat. Interestingly, various types of ABR were isolated from raw milk, and MDR should be monitored regularly. *Klebsiella* spp. presented with various MDR patterns. In addition, GEN and SXT were the most commonly resistant antibiotics. More comprehensive studies should be conducted to explain the confounding risk factors and molecular mechanisms underlying ABR and MDR. In addition, there is a need to establish an effective ABR and MDR surveillance system in the dairy sector in this region.

## Authors’ Contributions

SS: Collected the samples, collected data, performed the milk composition analysis, and drafted the manuscript. PS: Performed sample size calculation, analyzed data, and drafted the manuscript. WM: Drafted the manuscript, analysed data, and edited the manuscript. TB: Performed the milk composition analysis. VN, AKP, and MLP: Drafted the manuscript, edited the manuscript, and interpreted the data. All authors have read, reviewed, and approved the final manuscript.
